# Population neuroimaging: generation of a comprehensive data resource within the ALSPAC pregnancy and birth cohort

**DOI:** 10.12688/wellcomeopenres.16060.1

**Published:** 2020-08-28

**Authors:** Tamsin H. Sharp, Nancy S. McBride, Amy E. Howell, C. John Evans, Derek K. Jones, Gavin Perry, Stavros I. Dimitriadis, Thomas M. Lancaster, Luisa Zuccolo, Caroline Relton, Sarah M. Matthews, Thomas Breeze, Anthony S. David, Mark Drakesmith, David E. J. Linden, Tomas Paus, Esther Walton

**Affiliations:** 1 MRC Integrative Epidemiology Unit, Population Health Sciences, University of Bristol, Bristol, BS8 2BN, UK; 2 Cardiff University Brain Research Imaging Centre (CUBRIC), School of Psychology, Cardiff University, Cardiff, CF24 4HQ, UK; 3 ALSPAC, Population Health Sciences, Bristol Medical School, University of Bristol, University of Bristol, Bristol, BS8 2BN, UK; 4 Institute of Mental Health, University College London Medical School, London, W1T 7NF, UK; 5 MRC Centre for Neuropsychiatric Genetics and Genomics, Division of Psychological Medicine and Clinical Neurosciences, Cardiff University, Cardiff, CF24 4HQ, UK; 6 Bloorview Research Institute, Holland Bloorview Kids Rehabilitation Hospital and Departments of Psychology and Psychiatry, University of Toronto, Ontario, M4G 1R8, Canada; 7 Department of Psychology, University of Bath, Bath, BA2 7AY, UK

**Keywords:** neurodevelopment, neuroimaging, brain morphology, brain function, ALSPAC, population health, birth cohort, MRI

## Abstract

Neuroimaging offers a valuable insight into human brain development by allowing *in vivo* assessment of structure, connectivity and function. Multimodal neuroimaging data have been obtained as part of three sub-studies within the Avon Longitudinal Study of Parents and Children, a prospective multigenerational pregnancy and birth cohort based in the United Kingdom. Brain imaging data were acquired when offspring were between 18 and 24 years of age, and included acquisition of structural, functional and magnetization transfer magnetic resonance, diffusion tensor, and magnetoencephalography imaging. This resource provides a unique opportunity to combine neuroimaging data with extensive phenotypic and genotypic measures from participants, their mothers, and fathers.

## Introduction

Population neuroscience, the interface between epidemiology and neuroscience, aims to identify environmental and genetic factors associated with brain health across the lifespan ^1, 2^. Key to the development of this field is the recruitment of large, representative samples of participants, with deep phenotyping on a wide range of physiological, environmental and genetic measures. Over the last decade, the inception of large-scale population based studies ^3– 10^, and those embedded in existing cohorts ^9, 11^, has generated a rich source of neuroimaging data drawn from the general population.

Here we present three such studies nested within The Avon Longitudinal Study of Parents and Children (ALSPAC) ^12– 14^. In addition, we describe the pipelines and quality control (QC) measures used to generate image-derived phenotypes (IDPs) from T _1_-weighted structural magnetic resonance imaging (MRIs), designed to be useful to researchers in fields of neuroscience and beyond. ALSPAC is a prospective pregnancy and birth cohort, which enrolled a total of 14,062 pregnant women living in South-West England in the 1990s. Mothers, fathers and children have been followed up for the last 28 years, and data collection is ongoing. The study has collected genetic, epigenetic, and a wealth of phenotypic and environmental measures in a broad range of health, social and developmental domains. With such an abundance of data, ALSPAC provides a unique opportunity to identify factors for optimal neurodevelopment in the general population.

## Materials and methods

The Avon Longitudinal Study of Parents and Children (ALSPAC: formerly the Avon Longitudinal Study of Pregnancy and Childhood) is a pregnancy and birth cohort established to identify the factors influencing child health and developmental outcomes. All pregnant women residing in the county of Avon, South West of England, with an expected delivery date from 1 ^st^ April 1991 to 31 ^st^ December 1992 were invited to participate ^15^. A total of 14,541 pregnancies were initially enrolled (for details, see http://www.bristol.ac.uk/alspac/); of these 68 have no known birth outcome, 195 were twin, 3 were triplet and 1 was quadruplet, overall accounting for 14,676 known foetuses. This resulted in 14,062 live births, of whom 13,988 were alive at 1 year of age. A second wave of enrolment invited all eligible children and those not originally recruited to participate, which resulted in a total of 15,247 pregnancies ^13^. Since recruitment children and their parents have been followed up with questionnaire and clinical assessment data collected at regular intervals. Additionally, there is a detailed biobank, which includes biological samples, genetic and epigenetic measures. Full details on the cohort profile, representativeness, and phases of enrolment have been extensively documented ^15^. Further information regarding the ALSPAC cohort can be located on the study website, which includes a searchable data dictionary. Written informed consent was collected for all participants in line with the Declaration of Helsinki ^16^. Ethical approval for all neuroimaging sub-studies described below were obtained from the ALSPAC Ethics and Law Committee and the Local Research Ethics Committees (North Somerset & South Bristol Research Ethics Committee: 08/H0106/96) and participants provided written consent.

## Imaging sub-studies

### Overview

**Figure 1.**
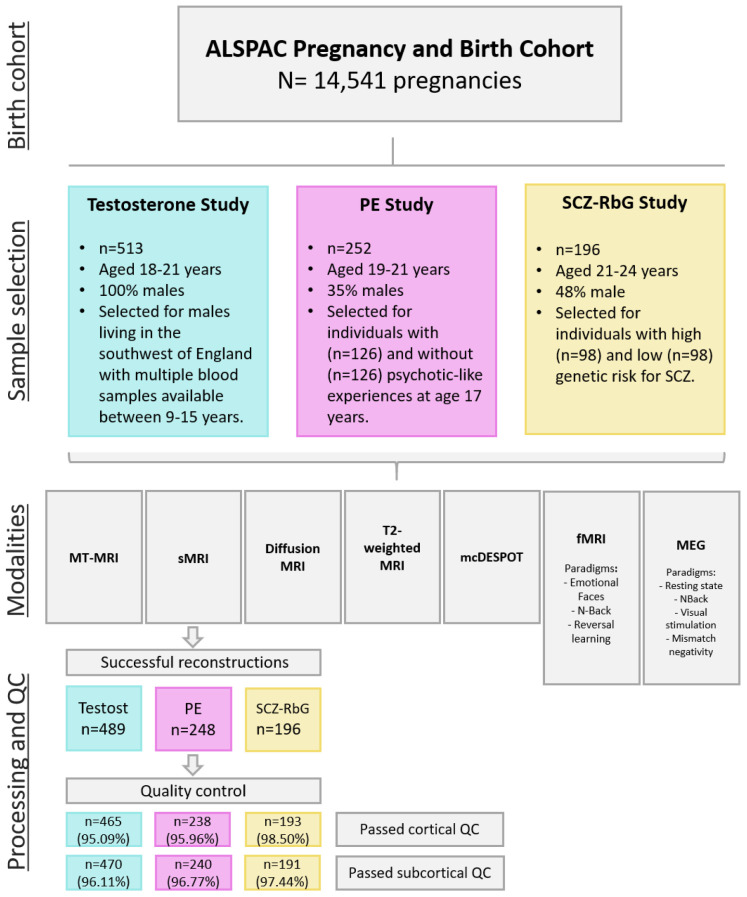
Flow chart depicting the sampling of each neuroimaging sub-study within ALSPAC, modalities acquired, and processing pipeline of structural MRIs. IDP: image-derived phenotypes, MT-MRI: magnetization transfer MRI, mcDESPOT (multi-component driven equilibrium single-pulse observation of T1 and T2). sMRI: structural MRI, DTI: diffusion tensor imaging, fMRI: functional MRI, MEG: magnetoencephalography, QC: quality control.

**Figure 2.**
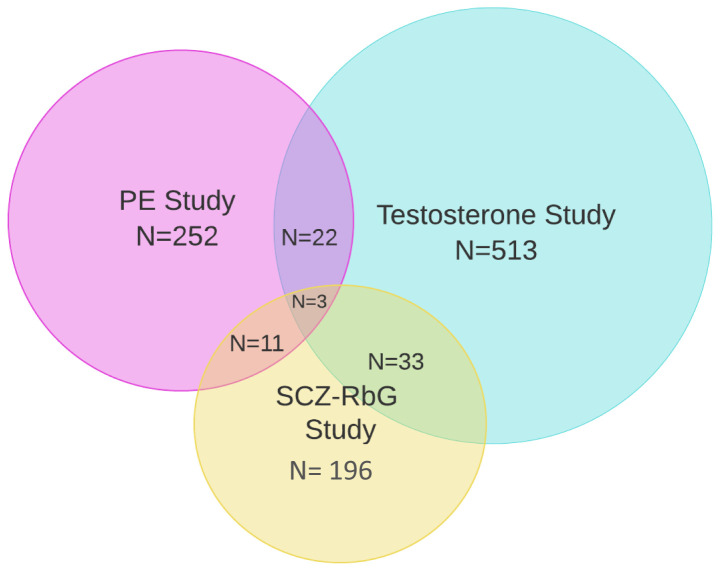
Venn diagram depicting participant overlap within the three ALSPAC-MRI sub studies. A total of 22 individuals participated in both the Testosterone and PE studies, 33 individuals participated in both the Testosterone and SZC-RbG studies, 11 individuals in both the PE and SCZ-RbG studies, and three individuals participated in all. PE: psychotic experiences, SCZ-RbG: schizophrenia-Recall-by-Genotype.

Between the ages of 18 to 24 years, a subset of ALSPAC offspring were invited to participate in three different neuroimaging studies; the ALSPAC Testosterone study (n= 513, mean age at attendance 19.62 years, range 18.00 to 21.50 years), the ALSPAC Psychotic Experiences (PE) study (n=252, mean age at attendance 20.03 years, range 19.08 to 21.52 years), and the ALSPAC Schizophrenia Recall-by-Genotype (SCZ-RbG) study (n=196, mean age at attendance 22.75 years, range 21.12 to 24.55 years). Scanning protocols were harmonised across sub-studies where possible, and all data were acquired at Cardiff University Brain Research Imaging Centre (CUBRIC) on a 3 Tesla General Electric HDx (GE Medical Systems) using an 8-channel head coil. Study specific sample descriptives and imaging information are described below and in Table 1. Sampling strategies and participant overlap are depicted in Figure 1 and Figure 2.

**Table 1.** Sample descriptives by neuroimaging sub-study. Ethnicity: derived from maternal self-report of ethnicity (“White” or” Non-White”) and her partner’s ethnicity (“White” or “Non-White”). Handedness: assessed by maternal report at 42 months of age. IQ: assessed at 15 years of age using the Wechsler Intelligence Scale. PE: psychotic experiences, eTIV: estimated total intracranial volume. *Owing to missing data, some cells do not sum to complete sample size.

		Testosterone Study		Psychosis Study				Recall-by Genotype Study				Core ALSPAC sample	
	
**Sample size**		513		252				196					
**Selection** **criteria (N, %)**		Healthy males (513, 100%)		Participants with PE (126, 50%) and healthy controls (126, 50%)				Participants with high genetic risk for SCZ (98, 50.00%) and those with low genetic risk (98, 50.00%)				All pregnant women residing in Avon, UK, with a due date from April 1991 to Dec 1992	
	
				**No PE**		**PE**		**Low genetic risk**		**High genetic risk**			
**Age: years**	Mean (SD)	19.62	(0.04)	20.10	(0.002)	20.05	(0.002)	22.54	(0.07)	22.87	(0.08)		
**Sex: N (%)**	Male	513	(100.00)	49	(38.89)	39	(30.95)	46	(46.94)	46	(46.94)	7356	(51.73)
	Female	0	(0.00)	77	(61.11)	87	(69.05)	52	(53.06)	52	(53.06)	6864	(48.27)
**Ethnicity: N (%)**	White	456	(96.41)	109	(95.61)	107	(97.27)	91	(100.00)	91	(100.00)	11186	(94.19)
	Non-white	17	(3.59)	5	(4.39)	3	(2.73)	0	(0.00)	0	(0.00)	690	(5.81)
**Handedness:** **N (%)**	Right	295	(63.17)	75	(68.18)	81	(71.68)	58	(66.67)	61	(70.93)	6507	(65.23)
	Left	54	(11.56)	11	(10.00)	5	(4.42)	10	(11.49)	12	(13.95)	1102	(11.05)
	Mixed	118	(25.27)	24	(21.82)	27	(23.89)	19	(21.84)	13	(15.12)	2367	(23.73)
**IQ score**	Mean (SD)	98.80	(0.56)	99.51	(1.10)	95.12	(1.18)	96.29	(1.33)	98.93	(1.51)	94.36	(0.18)
**eTIV: cm ^3^**	Mean (SD)	17597.13	(59.78)	16179.01	(196.27)	15997.19	(165.72)	16361.49	(176.55)	16051.87	(229.59)		

## ALSPAC-Testosterone Study

### Sample description

The ALSPAC-Testosterone Study was initiated to test associations between pubertal sex hormones and brain development in males from a population-based sample. A subset of 513 male participants from ALSPAC were selected based on the availability of multiple (>3) blood samples obtained during early and mid-puberty (9, 11, 13, 15 and 17 years of age), and their current residence being within a 3-hour journey (1 way) of the scanning centre in Cardiff, Wales. Participants were accepted based on those who first responded to the invitation ^17^. Measures acquired from this study have been used to assess sex differences in corpus callosal structure ^18^, the association of stress throughout the life course on white matter integrity ^19, 20^ and structural properties of the cerebral cortex ^21^. In addition, measures derived from structural MRIs have been used as part of a replication sample assessing the moderating role of a polygenic risk score for schizophrenia (SCZ) on the association between cannabis use and reduced cortical thickness ^22^. Ethical approval for the study was obtained from the ALSPAC Law and Ethics Committee and the Local Research Ethics Committees (listed at http://www.bristol.ac.uk/alspac/researchers/research-ethics/); participants provided written informed consent for their participation in this sub-study (ALSPAC project ID B648).

### Scanner acquisition parameters

For each participant (n=513; 100% male), multimodal neuroimaging data were acquired on a General Electric 3T HDx scanner and included structural MRI (sMRI), Diffusion Tensor Imaging (DTI), Multi-Component Driven Equilibrium Single-Pulse Observation of T1 and T2 (mcDESPOT), magnetization transfer ** MRI (mtMRI) and functional MRI (fMRI).

### Structural MRI

During each structural imaging session coronal T _1 _scans were collected. Imaging parameters were as follows: 3D fast spoiled gradient echo (FSPGR) with 168–182 oblique-axial AC-PC slices, 1 mm isotropic resolution; flip angle = 20°; repetition time (TR) = 7.9 ms; echo time (TE) = 3.0 ms; inverse time (TI) = 450 ms; 1mm × 1mm x 1mm voxel size; slice thickness 1 mm; FOV (field of view) 256 × 192 mm matrix. T _1_- weighted scans took approximately 7.15 minutes each.

### Diffusion Tensor Imaging

Diffusion Tensor Imaging (DTI) data were obtained using a dual spin-echo, single shot echo-planar imaging sequence. A total of 30 gradient orientations and 3 non-diffusion weighted images (b = 0 s/mm ^2^) were acquired with the following parameters: resolution = 2.4 × 2.4 × 2.4 mm; FOV = 230 × 230 mm; acquisition matrix = 96 × 96; slice thickness = 2.4mm; number of slices = 60 (oblique-axial AC-PC); TR/TE = cardiac gated/87ms (effective); b = 1200 s/mm ^2^, T _1_ = 0; flip angle = 90°; number of excitations (NEX) = 1; parallel imaging acceleration factor (ASSET) = 2;30. Acquisition time was between 15 and 20 minutes.

### Multi-component driven equilibrium single-pulse observation of T1 and T2 (mcDESPOT)

Data were acquired using a 3D fast spoiled gradient recall (SPGR), with 8 T1-weighted SPGR, 2 inversion prepared SPGR and 15 T1/T2 weighted steady-state free precession images at a resolution of 1.72 × 1.72 × 1.70 mm ^23^. Acquisition time was approximately 20 minutes.

### Magnetization transfer MRI

Images were collected with a 3D SPGR sequence in the sagittal plane using the following parameters: resolution = 1.9 x 1.9 mm × 1.9mm; FOV = 240 × 240 mm; matrix =128 × 128; slice thickness = 1.9 mm; number of slices = 100; TR/TE = 26.7 ms/1.8 ms; TI = 0; flip angle = 5°, NEX = 0.75, ASSET = ON. Acquisition time was 4.27 minutes.

### Functional MRI

***Paradigm: dynamic faces***. During the functional MRI (fMRI) session, participants viewed short videoclips displaying ambiguous facial expressions (gestures such as nose twitching), angry facial expression or control stimuli (non-biological motion). The control stimuli were adapted from a study of Beauchamp and colleagues ^24^. The face stimuli were created as follows. A total of eight actors (four females) were filmed for the face movements. They were instructed to express different emotions starting from a neutral point. Short video-clips from the periods when the actors were not expressing the emotions but were nonetheless moving their face (e.g. twitching their nose, opening their mouth, blinking their eyes) were also extracted. A total of 20 video-clips were selected for the angry and ambiguous face movements respectively; four raters judged the intensity of each of emotion from those clips. The control stimuli consisted of black-and-white concentric circles of various contrasts, expanding and contracting at various speeds, roughly matching the contrast and motion characteristics of the faces and hands clips ^25^. Dynamic video clips of faces were presented as, compared with static faces, they elicit more robust responses in brain regions critical for face processing, such as the fusiform gyrus and amygdala, and engage a more elaborate network for face processing, including regions in the frontal cortex and along the superior temporal sulcus ^26^. The three viewing conditions were organized into 18s blocks (five “Ambiguous”, five “Angry”, nine control) for a total of 160 echo planar imaging (EPI) volumes in a single 6-minute fMRI run. Using this paradigm, probabilistic maps of the brain response to faces were created ^27^, global genetic contributions to the response estimated ^28^, and various developmental processes in population-based studies investigated ^8, 29– 32^.

### Acquisition parameters

Acquisition parameters were as follows: GE-EPI (AC-PC); resolution = 3.4 × 3.4 mm; FOV = 220 × 220 mm; matrix = 64 × 64; slice thickness = 2.4 mm; number of slices = 45; gap = 1 mm; TR/TE = 3000/35; TI = 0; flip angle = 90; NEX=1, acquisition time = 6.42. Slice ordering was interleaved (ascending).

## ALSPAC Psychotic Experiences (ALSPAC-PE)

### Sample description

The ALSPAC-PE Study was established to investigate the effects of subclinical psychosis on brain structure and function. A subsample of 4,323 participants aged 17–18 from the ALSPAC cohort were assessed for psychotic experiences (PE), using the psychotic‐like symptoms semi‐structured interview (PLIKS) ^33, 34^, administered by trained psychologists. A definite or suspected PE was confirmed using the clinical criteria of the Schedule for Clinical Assessment in Neuropsychiatry ^35^. Approximately 10% of those tested (n=433) were identified as having experienced at least one definite or suspected PE and were invited to participate in this study. Of these, 29.1% (n=126) were scanned. A total of 3,887 participants of the original pool did not show any symptoms of PE, and of these, 3.24% (n=126) agreed to undergo scanning. The control group were randomly selected until the number of participants in each group was balanced. The mean age at time of scanning was 20.10 (SE=0.002) years of age in the control group, and 20.05 (SE =0.002) in the PE group. A full description of the protocol and original findings have previously been reported ^36^.

The data acquired from this study have demonstrated that, in this population-based sample, PE are associated with region-specific reductions in gyrification of the left temporal gyrus and volume of the left occipital and right prefrontal gyri, but not white matter ^37^. Analysis of whole-brain structural connectomes demonstrated differences in both global and local topology in individuals with PE compared to those without ^38^. An additional study identified clusters of differences in measures of mean diffusivity and fractional anisotropy between participant groups ^39^.

 Ethical approval for this study was given by both the Cardiff University School of Psychology Ethics Committee and the ALSPAC Ethics and Law Committee, and informed consent was obtained from all participants (ALSPAC project ID B709).

### Scanner acquisition parameters

For each participant (n=252, 35% male, of which 126 with PEs and 126 controls) structural, diffusion, relaxometry, and on a slightly small subset of the sample, functional MR data were collected. Participants were instructed to get a typical night’s sleep before each scan, not to drink more than one alcoholic beverage, and to abstain from drinking coffee within 2 hours preceding each scan.

### Structural MRI

 T _1_-weighted structural images with a 1mm isotropic resolution were acquired using a FSPGR sequence (TR = 7.8 ms, TE = 3.0 ms, TI = 450 ms, flip angle = 20°, acquisition matrix = 256 × 192, zero-padded matrix = 256 × 256). T _2_-weighted whole brain scans were acquired using a coronal TSE sequence with the following parameters: TR=10000 ms for 3T, TR=9000 ms for 1.5T; TE=14 ms for 3T, TE=64 ms for 1.5T; FA=149 for 3T, FA=180 for 1.5T; Bandwidth=193 for 3T, Bandwidth=149 for 1.5T; voxel size same as for T _1 _scans; NEX=1 for the 3T, NEX=2 for the 1.5T. Acquisition of each volume took approximately 14.5 minutes. 

### Diffusion tensor imaging

Diffusion MRI comprising a cardiac-gated diffusion-weighted spin-echo echo-planar imaging sequence was used to obtain high angular resolution diffusion weighted images (HARDI). A total of 60 gradient orientations and 6 unweighted (b = 0 s/mm ^2^) images were acquired with the following parameters: TR = cardiac-gated, TE = 87 ms, acquisition matrix = 96 × 96, zero-padded matrix = 128 × 128), FoV = 230 × 230 mm. Following zero-padding, the reconstructed image resolution for the HARDI scans was 1.8 × 1.8 × 2.4 mm.

### Multi-component driven equilibrium single-pulse observation of T1 and T2 (mcDESPOT)

SPGR images across eight flip angles, one inversion recovery SPGR (IR-SPGR) and SSFP images across eight flip angles and two phase-cycling angles were acquired in a 3T GE HDx MRI system (General Electric Healthcare). A total of 25 images were acquired for each participant. All images were acquired in sagittal orientation with a slice matrix of 128 × 128 mm (1.72 × 1.72 mm resolution) with a minimum of 88 slices (slice thickness = 1.7 mm). Additional slices were added for some participants to ensure full head coverage. Sequence-specific parameters were as follows: SPGR: TE = 2.112 ms, TR = 4.7 ms, flip angles = 3°, 4°, 5°, 6°, 7°, 9°, 13° and 18°. IR-SPGR: TE = 2.112ms, TR = 4.7ms, IR = 450 ms, flip angle = 5°. SSFP: TE = 1.6 ms TR = 3.2 ms, flip angles of 10.59°, 14.12°, 18.53°, 23.82° 29.12° 35.29°, 45°, 60° and phase-cycling angles of 0° and 180°.

### Functional MRI data

***Acquisition parameters***. *T*_2_*-weighted gradient-echo echo-planar images along the axial plane parallel to the AC–PC line (TR = 2000 ms, TE = 30ms, flip angle = 75°, FOV = 240 × 240mm, resolution = 3.75 × 3.75 × 3.5 mm).

### Paradigm: Working Memory

A letter variant of the N-Back task was used. Participants were instructed to press a button with their index finger when the letter that was presented on the screen was identical to the one they saw *n* trials earlier, where *n* can be 1, 2, or 3. During 0-back testing, participants were instructed to press the button whenever the letter X was presented on the screen. Each condition was presented three times in a pseudorandom order in blocks of 14 items; each item lasted 2 s and was preceded by a 3 s written instruction on the screen. During each block, there were three correct combinations, giving a maximum of nine correct responses per condition. Including the instruction, each block was 31 s long, making the total duration of the N-Back task 372 s ^40^.

## ALSPAC-Schizophrenia Recall-by-Genotype

### Sample description

The SCZ-RbG study was established to understand the effects of genetic variants contributing to schizophrenia (SCZ) on brain developmental and behavioural outcomes, with a focus on decomposing effects according to specific biological pathways. A recall-by-genotype design, which increases power by sampling participants from the tails of genotypic distribution for a particular trait or risk factor ^41^, was utilised.

 All participants within ALSPAC were genotyped using the Illumina HumanHap550 quad chip genotyping platform and were subjected to standard quality control methods ^42^. A total of 8,653 offspring ALSPAC participants had genotypic data available for this study. Genetic risk scores (GRS) were calculated for each participant using methods outlined by the International Schizophrenia Consortium, based on results from the Psychiatric Genomics Consortium SCZ genome-wide association study ^43^. GRSs for schizophrenia (GRS _SCZ_), were derived for each individual using the “score” command in Plink (version 1.07) ^44^. This summed together the number of risk alleles (coded 0, 1 or 2) for each single nucleotide polymorphism (SNP), weighted using the logarithm of each SNP’s odds ratio for SCZ, using the Psychiatric Genomics Consortium summary statistics.

The GRS _SCZ_–based RbG was calculated based upon a GRS _SCZ_ generated from SNPs with a discovery GWAS training-set P ≤. 05 threshold, including approximately 5% of all imputed SNPs. This threshold was set as it as it was most predictive of SCZ liability in the primary GRS _SCZ_ analysis, using training data/summary statistics derived from the largest SCZ genome-wide association study (34,241 cases and 45,604 controls) ^43^. Of the total 8,365 individuals, 196 participants (low GRS _SCZ_ [n = 98]; high GRS _SCZ_ [n = 98]) completed a series of psychometric and neuroimaging paradigms that are robustly associated with the aetiology of SCZ. An additional 104 participants declined the invitation to participate. In agreement with previous studies, non-participation was associated with the high GRS _SCZ_ group (low GRS _SCZ_ [n = 40]; high GRS _SCZ_ [n = 64]) ^29^. Researchers were blind to which tail of the GRS _SCZ_ distribution each individual was selected from during both data collection and processing. In the final sample, the GRS _SCZ_ groups were matched for gender (low GRS _SCZ_: 52 female, 46 male; high GRS _SCZ_: 52 female, 46 male) ^42^. Ethical approval for the study was obtained from the ALSPAC Law and Ethics Committee and the local research ethics committees, and all participants provided written informed consent (ALSPAC project ID B1276). Results from this study have demonstrated differences in BOLD (blood oxygen level-dependent) signal between groups during reward processing in the ventral striatum and whole brain ^42^.

### Scanner acquisition parameters and MRI data analysis

Structural, DTI, and functional MR data were collected (n = 196 for sMRI, n=191 for DTI, n=190 for fMRI N-Back task, n=192 for fMRI reversal learning task, n=196 for MEG resting-state, n=199 for MEG N-Back task, and n=197 for participants completing three MEG mismatch negativity sessions). In most cases, two scan sessions (one for MEG and one for DTI/MRI/fMRI) were required to collect the complete imaging dataset. Participants were instructed to get a typical night’s sleep before each scan, not to drink more than one alcoholic beverage, and to abstain from drinking coffee within 2 hours preceding each scan.

### Structural scans

High-resolution 3-dimensional T _1_-weighted images were acquired using a 3D FSPGR with contiguous sagittal slices of 1 mm thickness (TR=7.9 s, TE = 3.0 ms, TI = 450 ms, flip angle = 20°, FOV = 256 × 256 × 176 mm to yield 1 mm ^3^ isotropic voxel resolution images) were collected for each participant.

### Structural MRI

T _1_-weighted structural scans were acquired using an oblique axial, 3D FSPGR with the following parameters: TR = 7.9 ms, TE = 3.0 ms, inversion time = 450 ms, flip angle = 20°, 1 mm isotropic resolution, with a total acquisition time of approximately 7 minutes.

### Diffusion tensor imaging

HARDI data were acquired using a cardiac-gated, peripherally gated twice-refocused spin-echo EPI sequence. A total of 60 gradient orientations and three non-diffusion weighted (b = 0 s/mm ^2^) images were acquired with effective TR/TE of 15R-R intervals/87ms, FoV = 230 × 230 mm, acquisition matrix = 96 × 96, zero-padded matrix = 128 × 128. Following zero-padding, the reconstructed image resolution for the HARDI scans was 1.8 × 1.8 × 2.4 mm. Sets of 60 contiguous 2.4-mm thick axial slices were obtained, with diffusion-sensitizing gradients applied along 30 isotropically distributed gradient directions (b = 1,200 s/mm ^2^).

### Multi-component driven equilibrium single-pulse observation of T1 and T2 (mcDESPOT)

SPGR images across eight flip angles, one inversion recovery SPGR (IR-SPGR) and SSFP images across eight flip angles and two phase-cycling angles were acquired. A total of 25 images were acquired for each participant. All images were acquired in sagittal orientation with a slice matrix of 128 × 128 mm (1.72 × 1.72 mm resolution) with a minimum of 88 slices (slice thickness = 1.7mm). Additional slices were added for some participants to ensure full head coverage. Sequence-specific parameters were as follows: SPGR: TE = 2.112ms, TR = 4.7ms, flip angles = 3°, 4°, 5°, 6°, 7°, 9°, 13° and 18°. IR-SPGR: TE = 2.112ms, TR = 4.7ms, IR = 450ms, flip angle = 5°. SSFP: TE = 1.6ms TR = 3.2ms, flip angles of 10.59°, 14.12°, 18.53°, 23.82° 29.12° 35.29°, 45°, 60° and phase-cycling angles of 0° and 180°.

### fMRI data

***Acquisition parameters***. Gradient echoplanar imaging data were acquired for each participant using the following parameters: 35 slices, slice thickness = 3 mm/1 mm gap, acquisition matrix = 64 × 64; FOV = 220 mm, TR = 2000 ms, TE = 35 ms, flip angle = 90°, ASSET factor; 2. All functional images were first motion scrubbed, where TRs with a frame wise displacement of greater than 0.9 were removed. A total of 354 volumes (12 minutes) for the reversal learning and 265 volumes (9 minutes) for the N-Back study were acquired.

### Paradigm: N-Back working memory task

Participants performed a cued sequence production task, responding to visually cued sequences by generating responses using their right-hand on a fibre-optic response box. Responses were made using four fingers of the left hand (the thumb was excluded). Visual cues were presented as a series of Arabic numbers from 1 to 4. Each number was mapped to one of the four buttons on the response box. N-Back experiments were designed with three load levels (0, 1 and 2) following the same order during the 6 runs. The total number of presented numbers for each N-Back level per run was 10 (resulting in 10 responses for 0-back level, nine response for 1-back level and eight response for the 2-back level). The sequence of presented numbers was generated via a randomisation procedure for each participant. Every block started with a label (‘0-back’,’1-back’,’2-back’) to notify the participant of the current N-Back level. During the task, participants were not informed about their performance.

Each number (trial) was presented for 2 s, separated by an inter-trial interval (ITI) lasting maximum 3 s, not including any time remaining from the previous trial. The duration of each block/run was fixed. Responses and reaction times to each stimulus were for subsequent analysis of behavioural performance. Participants completed six runs of each of three conditions. The total duration of the experiment was approximately 10 minutes, with 9 minutes the actual block/run time. Each block/run included 15 slices with a total duration of 30 s.

Participants had performed the same task earlier on the same day as part of a MEG acquisition, and so were already familiar and practiced with the task when performing during the MRI acquisition. Stimulus presentation was performed by MATLAB version 7.6 (MathWorks, Natick, MA) using the Psychophysics Toolbox version 3 (also functional in GNU Octave). Key-press responses were collected using a fibre-optic button box supplied by NATA Technologies (Coquitlam, BC).

### Paradigm: reversal learning

Participants learned to choose one of two simultaneously presented colours (“blue” and “green”) by receiving monetary reward for correct choices and monetary punishment for wrong choices (e.g., +1 pence (p] for “blue” and −1p for “green”). After 7–11 trials, reward/punishment contingencies were reversed so that the previously rewarded colour was now punished and vice versa. Participants were instructed to maximize their earnings during the learning session, which consisted of 12 reversal episodes in total (108 choice trials). Within each reversal episode we included either one or two probabilistic error trials, in which “wrong”-feedback was given for correct choices, even though the reward contingencies had not changed. At the start of each choice trial, participants were presented with a response cue consisting of two white frames surrounding the colours and prompting the participants to press the left or right button on a response box to choose one colour. Response feedback (choice outcome) was given subsequently using a centrally presented white “smiley” (correct choice) or red “frowny” (incorrect choice) face and an earnings counter changing incrementally by ±1 p. In trials following reversal or probabilistic error events, i.e., in those trials used for fMRI analysis, response cues and feedback stimuli were presented with a jittered duration (cue: 4–8 s, mean 5.5 s; feedback: 0.75 s followed by 3–7 s (mean 4.5 s)) ITI. To reduce scanning time, in all other standard trials a fixed stimulus duration was used (cue = 2 s, feedback = 0.75 s). ITIs showed the two colours without response cue or feedback and were 0.5-s long after standard trials, and between 4 and 8 s (mean 5.5 s) after probabilistic errors and reversals.

### Magnetoencephalography (MEG)

***Acquisition parameters***. Whole-head MEG data were acquired using a 275-channel CTF axial gradiometer system sampled at 1200 Hz. An additional 29 reference channels were recorded for noise cancellation purposes. Vertical and horizontal electrooculography were also collected as bipolar recordings.

All experiments were run on MATLAB using the Psychophysics Toolbox ^45, 46^. Stimuli for all experimental paradigms were presented using a Mitsubishi Diamond Pro 2070 CRT monitor viewed through a hole cut into the shielded room. All displays were presented at 1024 × 768 resolution and 100-Hz refresh rate.

### Paradigm: resting state

Participants underwent a 5-minute resting state recording. Participants were instructed to keep their eyes open and to fixate on a centrally presented red square (approximately 0.2° in width).

### Paradigm: N-Back

Participants performed a cued sequence production task that was identical to that performed in the fMRI session.

### Paradigm: visual stimulation

Participants undertook 100 trials of visual stimulation with a grating stimulus. The stimuli were stationary, vertically oriented, luminance-defined, square-wave gratings with a spatial frequency of three cycles/°. Each stimulus was masked by a square window measuring 8 × 8° and presented centrally at maximum contrast on a mean luminance (26.5 cd/m ^2^) grey background.

Each trial consisted of a 2000-ms baseline period, followed by presentation of the stimulus for a random duration between 1500 and 2000 ms, followed by a 1000-ms response period, resulting in a total trial time between 4500 and 5000 ms. During each trial a red square (approximately 0.2° in width) was present continuously, and participants were instructed to maintain fixation on the square throughout. To encourage participants to maintain attention to the stimuli, they were instructed to respond to stimulus offset from the screen by pressing a single button with the index finger of their right hand as rapidly as possible.

### Paradigm: mismatch negativity

Participants undertook a mismatch negativity paradigm based on the ‘Optimum-1’ paradigm of Näätänen *et al.* ^47^. Participants listened to a sequence of tones that alternated between a standard tone and one of a number of deviant tones with stimulus onset asynchrony of 300 ms ^5^.

The standard stimulus was a harmonic tone composed of three sinusoidal partials of 500, 1000, and 1500 Hz, and was 75 ms in duration. The intensity of the second and third partials was lower than that of the first partial by 3 and 6 dB, respectively. The stimuli were presented via headphones at 60 dB above the individual participant's hearing threshold with equal phase and intensity at both ears.

The deviant tones differed from the standard tone by one of the following properties: frequency, duration, intensity, perceived sound-source location, or by having a gap in the middle of the tone. For frequency deviants, half were 10% higher in frequency from the standard and the other half were 10% lower. For intensity deviants, half were +10dB relative to the standard and half were -10dB. For sound-source deviants, an interaural delay of 800µs was introduced to the right ear half of the deviants, and for the left ear for the other half. The duration deviant had a duration of 25 ms. The gap deviant was created by leaving a silent gap of 7 ms in the middle of the stimulus. Deviant tones were otherwise identical to the standard tones.

The tones were presented for 15 minutes, split into 5-minute blocks with short breaks between. The order of deviant tones was randomised for each participant subject to the constraint that successive deviants were always of a different type. 

## Extraction of image-derived phenotypes from structural MRIs

T _1_-weighted images were processed using the automated FreeSurfer brain imaging software package (Version 6.0.0) via the ‘recon-all’ command including the -qcache flag. Processing includes an automated pipeline of removal of non-brain tissue, voxel intensity correction for B _1_ field inhomogeneities, segmentation of voxels into white matter, grey matter or cerebral spinal fluid, and generation of surface-based models of white and grey matter. Each vertex within the cortical ribbon is automatically assigned a label based on a predefined atlas, and parcellated into 34 cortical regions. Each voxel within the normalised brain is then assigned 1 of 42 labels, which includes 8 subcortical regions ^48^.

Reconstructed images were subjected to QC measures following the ENIGMA consortium structural image processing protocol ^49^. This included blinded visual inspection by two independent reviewers of the cortical external parcellation, cortical internal parcellation and subcortical segmentation via html brainmap outputs. The quality of cortical parcellation was determined by inspecting both lateral and medial snapshots of pial surface reconstructions, and internal slices through the brain. Subcortical images, and their segmentation, were assessed using the same protocol. Images were rated as “pass”, “moderate”, or “fail” quality for cortical parcellation and subcortical segmentation separately. Fail level scoring was defined as the presence of motion or other artefacts that significantly compromised image quality. Images from participants with substantial deviation from average neuroanatomy (e.g., volume of ventricles, skull shape) were identified and discussed with two additional reviewers to determine if this affected the overall parcellation or segmentation quality, and rated accordingly. No manual editing was applied to the reconstructed images.

Histograms of FreeSurfer output were assessed to confirm normal distribution of all extracted measures. Participants with thickness, surface or volumetric measures that deviated ± 2.698*SD were identified and closely inspected in the brainmap images. If a participant was identified as an outlier, but visual inspection confirmed accurate parcellation/segmentation, this did not affect their overall rating.

Within the three sub-studies the following number of reconstructed images failed quality control: Testosterone study: 5.0% (n=24) of cortical and 3.7% (n=18) of subcortical scans, PE Study; 4.03% (n=10) of cortical and 1.61% (n=4) of subcortical scans, SCZ-RbG Study; 2.03% (n=4) of cortical and 3.05% (n=6) of subcortical scans. Overlap of failure of cortical and subcortical quality control was as follows; Testosterone Study 20.85% (n=5), PE Study 0% (n=0), and SCZ-RbG Study 100% (n=4).

Image derived phenotypes of global, cortical and subcortical measures are available in csv format. In addition, all files produced from the recon-all pipeline are available for whole brain analyses. Results of the visual inspection of FreeSurfer output are also available to researchers, and we recommend exclusion of participants with a “fail” status, and the inclusion of sensitivity analyses with the removal of participants with a “moderate” quality image.

## Data availability

### Underlying data

ALSPAC data access is through a system of managed open access. To access the ALSPAC data included in this data note, and all other ALSPAC data, please follow the steps below.

1. Please read the ALSPAC access policy ( http://www.bristol.ac.uk/media-library/sites/alspac/documents/researchers/data-access/ALSPAC_Access_Policy.pdf) which describes the process of accessing the data and samples in detail, and outlines the costs associated with doing so.
2. You may also find it useful to browse our fully searchable research proposals database ( https://proposals.epi.bristol.ac.uk/?q=proposalSummaries), which lists all research projects that have been approved since April 2011.
3. Please submit your research proposal ( https://proposals.epi.bristol.ac.uk/) for consideration by the ALSPAC Executive Committee. You will receive a response within 10 working days to advise you whether your proposal has been approved.

Data are available in the following formats: structural MRI (NifTI, defaced), mcDESPOT (raw DICOM), functional MRI (raw DICOM), DTI (raw DICOM). Image derived phenotypes extracted from the sMRIs using FreeSurfer and QC results are available in csv format. Full details regarding the numbers of available datasets across modalities for each ALSPAC sub-study are described in Table 2.

**Table 2.** Numbers of available datasets across modalities for each ALSPAC neuroimaging sub-study. MRI: magnetic resonance imaging, sMRI: structural MRI, fMRI: functional MRI, DTI: diffusion tensor imaging, mcDESPOT (multi-component driven equilibrium single-pulse observation of T1 and T2), FSPGR: fast spoiled gradient echo, PE: psychotic experiences, SCZ-RbG: schizophrenia-Recall-by-Genotype, NifTI: Neuroimaging Informatics Technology Initiative, CSV: comma-separated values, DICOM: Digital Imaging and Communications in Medicine. IDPs (image derived phenotypes) were extracted from sMRIs using FreeSurfer (version 6.0).

	sMRI	sMRI	fMRI	fMRI	fMRI	mcDESPOT	DTI
	T1-type FSPGR	IDP	Faces	N-Back	Reversal Learning		
**Testosterone Study**	504	489	474			489	500
**PE Study**	252	249		214		248	250
**SCZ-RbG Study**	195	195		192	192	183	189
**Format**	NifTI	CSV	raw DICOM	raw DICOM	raw DICOM	raw DICOM	raw DICOM
