# Supplementary material for: Population neuroimaging: generation of a comprehensive data resource within the ALSPAC pregnancy and birth cohort
Source: Wellcome Open Res. 2020 Aug 28;5:203. [Version 1] doi: 10.12688/wellcomeopenres.16060.1 (PMC7531050; doi:10.12688/wellcomeopenres.16060.1)
Supplement: Peer review history

# Peer review: Reviewer response for version 1

**DOI:** 10.21956/wellcomeopenres.17616.r40209

**Published:** 2020-10-01

**Authors:** Kirsten A. Donald

**Affiliations:** 1 Division of Developmental Paediatrics, Department of Paediatrics & Child Health, Red Cross War Memorial Children's Hospital, University of Cape Town, Cape Town, South Africa

This manuscript presents a summary of three neuroimaging substudies nested in the ALSPAC cohort and focused on young adults between the ages of 18-21 years. The studies include a more general population-based study in males only related to understanding the role of testosterone in adolescence on brain development (n=513), a more targeted study investigating brain structure and function amongst young adults who have suffered a psychosis like experience/episode and then a highly targeted investigation of high vs low genetic risk for schizophrenia and brain structure and function in young adulthood. This is a well written study which is potentially a very useful resource for internal and external researchers to support the broader use of the data.

 The hypothesis-based aims of the three studies mean that the potential to use this data for answering other questions may be limited. The fact that the samples are so highly selected should have some reflection in the abstract. Given that the rationale for the paper is to describe the different data sets in a manner that may allow them to be used for other aims, a more detailed discussion of how the datasets overlap not only in terms of numbers, but also acquisition sequences, processing approaches across different modalities (not just the structural). Within the descriptions of the different studies, detail is given about acquisition sequences (and functional MRI task-paradigms) as well as a variable amount of detail about QC approaches. Having more detail about what domains the paradigms were attempting to establish across the different cohorts would be useful for outside researchers to understand how the task-based fMRI data may potentially be used. More closely aligning the way the studies are reported in the manuscript (including how much information is offered regarding sample selection, sequences acquired, acquisition protocols, QC, results summary and potential limitations of the datasets) would also make it easier to make sense of how the very different studies might intersect. A discussion section which synthesizes this information in a narrative form would be very helpful. This can include potential strategies for using these data as well as clear discussion of limitations.

Are sufficient details of methods and materials provided to allow replication by others?

Partly

Is the rationale for creating the dataset(s) clearly described?

Partly

Are the datasets clearly presented in a useable and accessible format?

Partly

Are the protocols appropriate and is the work technically sound?

Yes

Reviewer Expertise:

Paediatric neurology and population neuroimaging

I confirm that I have read this submission and believe that I have an appropriate level of expertise to confirm that it is of an acceptable scientific standard, however I have significant reservations, as outlined above.

# Peer review: Reviewer response for version 1

**DOI:** 10.21956/wellcomeopenres.17616.r40208

**Published:** 2020-09-18

**Authors:** Koen Bolhuis

**Affiliations:** 1 Department of Child and Adolescent Psychiatry/Psychology, Erasmus MC-University Medical Center-Sophia Children’s Hospital, Rotterdam, The Netherlands; 2 Department of Psychiatry, Amsterdam University Medical Centre - location AMC, Amterdam, The Netherlands

Many thanks for giving me the opportunity to review this paper. This study describes three neuroimaging (MRI) projects nested in the population-based ALSPAC birth cohort. The three individual projects include the ALSPAC-Testosterone Study (n=513; males only), ALSPAC Psychotic Experiences Study (n=252) and ALSPAC Schizophrenia Recall-by-Genotype (n=196). This study describes how brain imaging data were collected for these separate projects and what quality control procedures were applied. In addition, it briefly discussed the results from these studies and how researchers can get access to the data. The paper reads very nicely and coherently, and QC protocols are presented in great detail. It is also very clear from the table how many individuals had complete data available for the specific imaging modalities datasets. However, I would find it interesting to read more about these sub-project taken together and what are the future directions of neuroimaging in ALSPAC, perhaps in a separate/final paragraph of the paper(?)

 The study described the individual scanning and QC protocols, and that they were harmonised across sub-studies where possible. I would be interested to read which parts of the protocol could not be harmonised. And could this have had any effects on the results and their interpretation?

 As shown in the Venn diagram, there is little overlap of participants from the 3 sub-projects (naturally, because of the 3 quite different hypotheses and research aims). However, has there been any effort made to make these number bigger? It would have given so many opportunities to study longitudinal adolescent/early-adulthood brain development.

 In addition, I think it would be interesting to read something about the future directions of neuroimaging studies in ALSPAC. Are there any research plans of the authors using these already available dataset? Is there a way to harmonise them more in order to more fully benefit from the wealth of ALSPAC data?

 Relatedly, are there new ALSPAC-MRI sub-projects planned?

 Also, I've noticed that not all of the imaging modalities of the 3 separate projects have been published about in peer-reviewed articles. For example, for the SCZ-RbG study, only structural and functional MRI (reversal learning paradigm) have been studied. Are there any plans to look at the other modalities too? Or have these resulted in null findings?

 Minor point: in Table 1, the authors wrote "Psychosis Study", but perhaps PEs study would fit better with the rest of the article.

Are sufficient details of methods and materials provided to allow replication by others?

Yes

Is the rationale for creating the dataset(s) clearly described?

Partly

Are the datasets clearly presented in a useable and accessible format?

Yes

Are the protocols appropriate and is the work technically sound?

Yes

Reviewer Expertise:

Child and adolescent psychiatry, epidemiology, psychosis risk

I confirm that I have read this submission and believe that I have an appropriate level of expertise to confirm that it is of an acceptable scientific standard, however I have significant reservations, as outlined above.

# Peer review: Reviewer response for version 1

**DOI:** 10.21956/wellcomeopenres.17616.r40211

**Published:** 2020-09-16

**Authors:** Linnea Karlsson

**Affiliations:** 1 FinnBrain Birth Cohort Study, Turku Brain and Mind Center, Department of Clinical Medicine, University of Turku, Turku, Finland; 2 Centre for Population Health Research, Turku University Hospital and University of Turku, Turku, Finland; 3 Department of Psychiatry, Clinic of Adolescent Psychiatry, Turku University Hospital and University of Turku, Turku, Finland

This paper describes three brain imaging (MRI) projects nested in the famous ALSPAC birth cohort. The emphasis is on imaging methodology of these projects, i.e. ALSPAC-Testosterone Study (n=513; males only), ALSPAC Psychotic Experiences Study (n=252) and ALSPAC Schizophrenia Recall-by-Genotype (n=196). The paper is very coherent and describes clearly the scanning and image processing protocols.

 The quality control (QC) dimension that is highlighted in the abstract/overview is mentioned only rather briefly by citing the well-known ENIGMA consortium protocol. Accomplishing long-term cohort follow-ups and collecting data from multiple sources using demanding methods and techniques as here, is to be complimented.The sample sizes are large or largish for the field, while I read no comments on power issues re: the possible main outcomes.

 I understand that the focus in this paper is in illustrating the methods of these projects e.g. for the purposes of the projects, data sharing and other collaborations. However, I still miss more information on the rationale, aims and expected results or hypotheses (if any). It would be interesting to hear, why these domains were selected as foci here. Which metrics are the most interesting ones at this stage? What were the most burning questions to be answered by these studies? Or was the idea more of collecting an image bank from a population based cohort around certain themes without having highly focused questions in mind? both approaches are ok, but a comment on this would be nice.

Are sufficient details of methods and materials provided to allow replication by others?

Yes

Is the rationale for creating the dataset(s) clearly described?

Partly

Are the datasets clearly presented in a useable and accessible format?

Yes

Are the protocols appropriate and is the work technically sound?

Yes

Reviewer Expertise:

birth cohorts, population health research, child and adolescent psychiatry, prenatal stress, early life stress, child development

I confirm that I have read this submission and believe that I have an appropriate level of expertise to confirm that it is of an acceptable scientific standard.
